# The Hidden Trigger of Migraine: The Role of Intranasal Mucosal Contact Points

**DOI:** 10.5152/eurasianjmed.2025.251057

**Published:** 2025-10-23

**Authors:** Murat Yaşar, İdris Kocatürk

**Affiliations:** 1 Department of Otorhinolaryngology, Kastamonu University Faculty of Medicine, Kastamonu, Türkiye; 2 Department of Neurology, Kastamonu Training and Research Hospital, Kastamonu, Türkiye

**Keywords:** Headache, migraine, nasal septum

## Abstract

**Background:**

Intranasal mucosal contact points (MCPs) can exacerbate primary headaches or give rise to secondary headaches. In this study, the prevalence of intranasal MCPs and their relationship with migraine features were investigated in patients diagnosed with episodic migraine.

**Methods:**

Fifty migraineurs were enrolled in the migraine group, and 50 without migraine in the control group in this retrospective study. Visual Analog Scale (VAS), Migraine Disability Assessment (MIDAS), and Headache Impact Scale (HIT-6) scores were retrieved from the patient files. Coronal and axial computed tomography sections were scanned, and intranasal MCP and anatomical variations within these were recorded. The prevalence of MCP was then compared across the 2 groups.

**Results:**

The patient group had a considerably higher prevalence of MCP than the control group (*P* = .018). No meaningful correlations were detected between mucosal contact and age, sex, migraine aura, menstruation, frequency of migraine attacks, duration of attacks, pain severity (VAS), or the effect of migraine on daily living (MIDAS) and quality of life (HIT-6).

**Conclusion:**

The findings indicate that an increased prevalence of migraine is associated with intranasal mucosal contact, which occurs particularly between the middle concha and septum. Further clinical studies researching the role of intranasal mucosal contact in migraine are now needed.

**Main Points**

- Intranasal mucosal contact points (MCPs) are found to be significantly more prevalent in migraine patients when compared to control groups.
- The most common intranasal MCP is located between the middle concha and the septum.
- The study suggests that intranasal MCP may trigger migraine attacks but does not influence their characteristics.

## Introduction

Migraine is a ubiquitous, debilitating neurological disease and a significant cause of loss of workforce productivity in adulthood. Headache is the primary symptom of migraine headaches.^1^^,^^2^ The condition is classified as episodic or chronic.

Migraine attacks are defined as severe pain lasting 4-72 hours, generally unilateral, and accompanied by nausea and/or vomiting and photophobia.^3^ In migraine patients, the Visual Analog Scale (VAS), Migraine Disability Assessment (MIDAS), and Headache Impact Test (HIT-6) can be used to assess pain severity, disability in relation to migraine, and the impact of headaches on daily activities, respectively.

Migraine pain has previously been linked to various nasal pathologies, such as septum deviation and particularly middle or superior concha anomalies. These nasal pathologies may cause contact between the nasal septum and the lateral nasal wall.^4^ The prevalence of endonasal changes and intranasal mucosal contact points (MCPs) in patients diagnosed with migraine ranges from 4% to 55%.^4^ Any contact point in the nasal cavity can be detected using sinonasal endoscopy and computed tomography (CT).^5^ Contact points can exacerbate primary headaches or represent a source of secondary headaches. Despite the absence of such evidence, intranasal mucosal contact headache was recently classified as a secondary condition in the International Classification of Headache Disorders (ICHD-3).^6^

Data concerning the effect of intranasal contact points on headaches in patients with migraine and the role of intranasal triggers in headache attacks are scarce.

In this study, the prevalence of intranasal MCP and their relationship with migraine features were ivestigated in patients diagnosed with episodic migraine.

## Materials and Methods

This study was conducted in accordance with the 2008 Declaration of Helsinki and with the approval of the Kastamonu University Clinical Research Ethical Committee (Approval no.: 2023-KAEK-142; Date: November 1, 2023).

**Figure 1.**
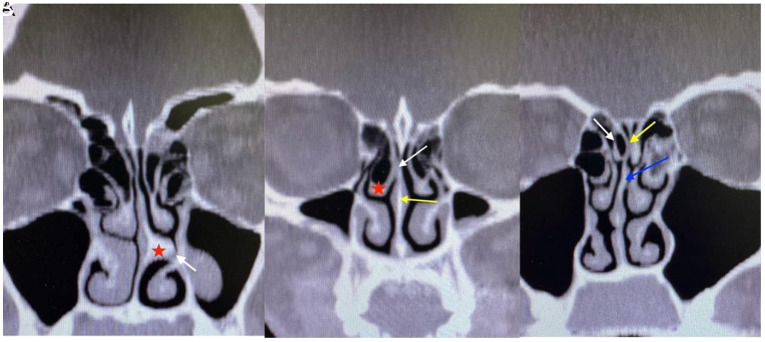
Coronal computed tomography scans show A: septal spur (red star), contact point (white arrow); B: middle concha bullosa (red star), contact point (white arrow), septum (yellow arrow); C: superior concha bullosa (white arrow), contact point (yellow arrow), septum (blue arrow).

This study involved a retrospective investigation of patients aged 18-65 years who underwent maxillofacial CT scans recorded in the Kastamonu Training and Research Hospital, Türkiye, system between October 2022 and October 2023, and who were diagnosed with migraine by the neurology clinic. Migraine was diagnosed by the ICHD-3. The VAS, MIDAS, and HIT-6 scores were recorded from the patient’s files. The control group consisted of individuals with no diagnosis of migraine, whose CT scans were obtained for clinical indications unrelated to neurological pathology. All scanning procedures were performed utilizing a CT scanner (GE Revolution EVO 128 slice, Chicago, IL, USA). The file scanning continued until 2 groups of 50 members each had been constituted. Informed consent in written form was obtained from the patients. Patients with any history of nasal or paranasal surgery, pansinusitis, nasal foreign body, nasal trauma, or nasal or paranasal masses were excluded. Patients with non-migraine headaches, such as tension-type headaches, cluster headaches, vascular headaches, nonvascular intracranial disorders, or psychogenic headaches, were also excluded. Additionally, patients with chronic migraine were excluded from the study. Fifty patients diagnosed with migraine, who followed up for at least 2 years were enrolled in the migraine group, and 50 individuals with no diagnosis of migraine and with no history of receipt of migraine treatment were included in the control group. Coronal and axial CT sections were scanned, and intranasal MCP between the structures constituting the septum and the lateral nasal wall and anatomical variations (Figure 1) causing mucosal contact within these were recorded.

### Statistical Analysis

All statistical evaluations were carried out using IBM SPSS Statistics software, version 22.0 (IBM Corp., Armonk, NY, USA). To assess whether the variables met the assumption of normal distribution, both histograms, quantile-quantile plots, and the Shapiro–Wilk test were utilized. Group comparisons for categorical variables were conducted using the chi-square test. Two-sample *t*-test and Mann–Whitney *U-*test were employed to analyze continuous variables with normal and non-normal distributions, respectively. Results with a *P*-value below .05 were interpreted as statistically significant.

## Results

The demographic characteristics and mucosal contact variables of the patient and control groups were compared, but no significant age difference was determined (*P* = .455). Gender distributions were also similar, with no significant difference in male-to-female ratios (*P* = .999). However, mucosal contact was observed to be significantly greater in the patient group (Group 1) in comparison to the control group (Group 2) (*P* = .018). Mucosal contact incidence was, as shown in Table 1, in 80% of patients in Group 1 and 56% of patients in Group 2.

The most common intranasal MCP was the “middle turbinate-septum,” observed in 32% and 22% in Group 1 and Group 2, respectively. However, the difference was insignificant (*P* = .134) (Table 1).

No meaningful difference was determined in age when the patient’s clinical and demographic variables were compared according to the presence of mucosal contact (*P* = .188). A comparison of groups indicates that there was no statistically meaningful difference in the gender distribution. Migraine aura was present in 60% of the group with mucosal contact and 80% of the non-contact group. However, this difference between the groups was not found to be statistically significant (*P* = .295). A family history of migraine was present in 40% of patients with mucosal contact and 60% of those without mucosal contact, but statistical analysis revealed no significant difference between the 2 groups (*P* = .302). Menstrual migraine was detected in 45.7% of the group with mucosal contact and in 50% of the non-contact group, with no statistically significant difference (*P* = .999). The difference was also not observed between the groups in regard to the number of migraine attacks per month (contact: 3 (2-4), no contact: 3 (2-4); *P* = .560). No statistically meaningful difference was also found regarding attack duration (hours/minutes) at 6 (5-21) hours in the mucosal contact group and 5.5 (5-24) in the non-contact group (*P* = .931). The VAS scores indicating the severity of pain were 8 (5.5-10) in the mucosal contact group and 8.5 (5.5-10) in the non-contact group, the difference being insignificant (*P* = .395). There was no statistically significant difference between the MIDAS scores of the groups; at 9.5 (0.75-29.50) in the mucosal contact group and 10.0 (6.0-20.25) in the non-contact group (*P* = .779). The HIT-6 scores were 62.0 (53.5-66.75) in the mucosal contact group and 68.0 (58.5-71.5) in the non-contact group, the difference also being insignificant (*P* = .106). To summarize, no significant associations were determined between the presence of mucosal contact and age, sex, migraine aura, family history, menstruation, number of migraine attacks, attack duration, pain scores (VAS), the effect of migraine on daily living (MIDAS), or quality of life (HIT-6) (Table 2).

The statistical analysis revealed that there was no statistically meaningful difference between Group 1 and Group 2 with regard to anatomical changes in MCP (*P* > .05). İn particular, widespread anatomical variations such as septum deviation, septal spur, and concha bullosa exhibited similar incidences between the groups. Although the incidence of changes such as medialized superior and middle concha was lower in the patient group, the difference was insignificant (Table 3).

## Discussion

Our study indicated a higher prevalence of MCP in patients suffering from migraine in comparison to the control group. However, in this study no meaningful associations were determined between the presence of mucosal contact and age, sex, migraine aura, family history, menstruation, number of migraine attacks, attack duration, pain scores (VAS), the effect of migraine on daily living (MIDAS), or quality of life (HIT-6).

Headache is a common presenting symptom with a large number of etiological causes. Endonasal anatomical abnormalities have long been regarded as 1 potential cause of headaches.^7^

The trigeminovascular theory in the pathophysiology of migraine is still valid. The trigeminal nerve innervates the anterior part of the head and face. Stimulation of the nerve triggers vascular dilation. The neurogenic inflammation that develops as a result then gives rise to migraine symptoms. The stimulation of some anatomical structures in the nasal cavity has been reported to be capable of stimulating the trigeminal nerve without nasal and paranasal mucosa inflammation and leading to the release of substance P.^8^^,9^ Headache probably commences due to the release of substance P from these MCP, as the trigeminal system is affected. Substance P leads to vasodilation, and amylin is a neurotransmitter for C fibers. The perception of pain is mediated by C fibers.^10^

The intranasal MCP is generally regarded as triggering a primary headache. Exposure to triggering variables can increase the risk and frequency of headache attacks. Contact points can also act as trigger points in migraineurs, making them more resistant to treatment.^5^ The number of MCP in this study was considerably higher in the migraine group than in the control group. However, no significant associations were determined between the presence of MCP and age, sex, migraine aura, family history, menstruation, numbers and durations of migraine attacks, pain severity (VAS), MIDAS, or HIT-6. This suggests that intranasal MCP may only trigger migraine attacks but have no effect on their character.

Sinonasal symptoms such as nasal pressure due to swelling and edema in the nose during migraine attacks, postnasal drip, nasal congestion, and itching suggest a relationship between intranasal structures and migraine.^11^^,^^12^ This is unsurprising since, in the case of trigeminal nerve activation, the inflammation caused is not limited to the first part of the nerve. Some studies have reported at least 1 sinonasal symptom in 46% of patients with migraine attacks.^12^

Contiguous nasal mucosal surfaces can trigger a migraine headache.^12^ Contact between endonasal mucosal surfaces leads to pain.^13^ This concept has led to surgical interventions directed toward intranasal anatomical variations causing mucosal contact in the treatment of migraine.^14^ Mucosal contact is thought to make migraine attacks resistant.^15^ However, no relationship was detected in the present study between the presence of MCP and the severity or duration of migraine attacks. This may be attributable to the exclusion of chronic migraine patients. Surgery directed toward MCP may represent a promising therapeutic alternative in migraine or tension-type headaches in carefully selected patients.^16^ Mariotti et al^7^ reported significant success with endoscopic nasal surgery applied to 33 patients with rhinogenic headaches, with improvement being observed in 84.8%. Novak et al^17^ reported complete remission in 78.5% of migraineurs who underwent surgery, and significant improvement in 11.5%. Another study of 21 migraine patients for whom surgery was decided based on local anesthetic tests and tomographic screening reported complete resolution of pain in 43%.^18^ However, there is no test, significant history, or CT parameter capable of predicting surgical outcomes in migraineurs.

An exact understanding of the relationship between migraine and MCP may facilitate the selection of the surgical technique. The number of studies involving detailed investigations of MCP in migraine patients is limited. In their study of 95 migraineurs, Lee et al^19^ detected septum deviation in 76.5%, septal spurs in 33.7%, concha bullosa in 48%, and paradoxical middle turbinate in 10.2%. They also reported that nasal contact point and concha bullosa surgery can successfully manage migraine. In their cohort study, Clerico et al^20^ determined septoturbinal mucosal contact in more than 49% of 355 subjects, with mucosal contact being most common at the level of the superior concha. In addition, they reported a correlation between mucosal contact between the superior concha and the septum, and an increased prevalence of migraine.^20^ The most common intranasal MCP among the migraine patients in the present study was between the middle concha and the septum (38%). This was followed by the superior concha and septum (28%) and the inferior concha and septum (24%). However, the incidences of widespread anatomical variations such as septal spur, septum deviation, middle concha bullosa, and superior concha bullosa were similar in the 2 groups.

The principal limitations of the present study are to be found in the relatively low number of patients included and the retrospective nature of the investigation. In addition, no distinction was made between active and inactive migraine, and no causal investigation was conducted between mucosal contact and migraine using the lidocaine test. The fact that patients with chronic migraine were excluded from the study also prevented a comprehensive inquiry concerning all migraine types.

Intranasal mucosal contact between the septum and conchae is common in migraineurs. Mucosal contact in this study was most common at the middle concha level. These findings showed that intranasal mucosal contact, particularly between the middle concha and nasal septum, is associated with an increased incidence of migraine. However, this increase was not statistically significant. Further well-designed controlled clinical studies are warranted to clarify the role of intranasal MCP in migraine pathophysiology, particularly focusing on the effects of surgical interventions targeting the middle and superior conchae in migraine management.

## Figures and Tables

**Table 1.** A Comparison of Demographic and Mucosal Contact Variables in the Patient and Control Groups

Variables	Group		* P *
	Patient (1) (n = 50)	Control (2) (n = 50)	
Age, years	37.88 ± 9.83	36.44 ± 9.38	.455
Gender, n (%)			
Female	42 (84.0)	42 (84.0)	.999
Male	8 (16.0)	8 (16.0)	
Mucosal contact, n (%)			
Present	40 (80.0)	28 (56.0)	**.018**
Absent	10 (20.0)	22 (44.0)	
Mucosal contact point, n (%)			
None	10 (20.0)	22 (44.0)	
Middle concha-septum	16 (32.0)	11 (22.0)	.134
Inferior concha-septum	9 (18.0)	4 (8.0)	
Superior concha-septum	8 (16.0)	6 (12.0)	
Inferior concha-septum, Superior concha-septum	3 (6.0)	2 (4.0)	
Middle concha-septum, Superior concha-septum	3 (6.0)	5 (10.0)	
Septum-posterior ethmoid cell	1 (2.0)	0 (0.0)	

**Table 2.** A Comparison of the Patient’s Demographic and Clinical Characteristics According to the Presence of Mucosal Contact

Variables	Mucosal Contact		* P *
	Present (n = 40)	Absent (n = 10)	
Age	38.80 ± 9.00	34.20 ± 12.51	.188
Gender, n (%)			
Female	33 (82.5)	9 (90.0)	.999
Male	7 (17.5)	1 (10.0)	
Migraine aura, n (%)			
Yes	24 (60.0)	8 (80.0)	.295
No	16 (40.0)	2 (20.0)	
Family history, n (%)			
Yes	16 (40.0)	6 (60.0)	.302
No	24 (60.0)	4 (40.0)	
Association with menstruation, n (%)			
Yes	16 (45.7)	4 (50.0)	.999
No	19 (54.3)	4 (50.0)	
Attack number (month)	3 (2-4)	3 (2-4)	.560
Attack durations (hours)	6 (5-21)	5.5 (5-24)	.931
VAS	8 (6.25-9)	8.5 (6.75-10.0)	.395
MIDAS	9.5 (0.75-29.50)	10.0 (6.0-20.25)	.779
HIT-6	62.0 (53.5-66.75)	68 (58.5-71.5)	.106

HIT-6, Headache Impact Scale; MIDAS, Migraine Disability Assessment; VAS, Visual Analog Scale.

**Table 3.** A Comparison of Anatomical Changes Observed in Mucosal Contact Points Between the Patient and Control Groups

Anatomical Changes	Patient (Group 1) (n = 50)	Control (Group 2) (n = 50)	* P *
Septal spur	9 (18.0)	9 (18.0)	.999
Septum deviation	12 (24.0)	12 (24.0)	.999
Middle concha bullosa	10 (20.0)	11 (22.0)	.806
Superior concha bullosa	10 (20.0)	10 (20.0)	.999
Inverted middle concha	2 (4.0)	5 (10.0)	.436
Medialized superior concha	2 (4.0)	2 (4.0)	.999
Medialized middle concha	2 (4.0)	4 (8.0)	.678
Excessive posterior ethmoid cell pneumatization	1 (2.0)	0 (0.0)	.999

## Data Availability

The data that support the findings of this study are available on request from the corresponding author.
